# Scarlatina, Vel Febris Rubra,—Scarlet, or Fever

**Published:** 1838-11-21

**Authors:** N. Chapman

**Affiliations:** Professor of the Theory and Practice of Physic, in the University of Pennsylvania


### THE MEDICAL EXAMINER.

DEVOTED TO MEDICINE, SURGERY, AND THE COLLATERAL SCIENCES.

**EDITED BY J. B. BIDDLE, M.D., M. CLYMER, M.D., AND W. W. GERHARD, M.D.**

No. 24.] PHILADELPHIA, WEDNESDAY, NOVEMBER 21, 1838. [Vol. I.

### SCARLATINA, VEL FEBRIS RUBRA,—SCARLET, OR FEVER.

*By* N. Chapman, M. D., *Professor of the Theory and Practice of Physic, in the University of Pennsylvania.*

[Reported for this Journal.]

This disease is so denominated, from the peculiar floridness of the skin incident to it. The first of these terms is Italian, and has been much objected to by the sticklers for a purely classical nomenclature, as a barbarous interpolation. For it, the second is one of many titles which have been proposed to be substituted. But neither this nor any other I have met with, has been happily selected—the whole being founded on a single appearance, and which is not a uniform one, or existing sufficiently expressive of the nature of the disease.

Doubtless, scarlatina is of modern date. Certain passages have been cited from some of the ancient writings, thought to refer to this, and its affiliated affections, which, on a more careful examination, will not bear such an interpretation. Its introduction into Europe, is commonly traced to an importation from Africa, and is said to have first broken out in Spain in 1610, whence it spread to Naples, where it prevailed eight years afterwards as an epidemic, with unexampled violence—fifty thousand persons having died of it in that city, and half a million in the Neapolitan and adjacent territories before its cessation.

By Sydenham, and subsequently Moreton, some account was given of it as prevailing in London in 1689. But so widely do their descriptions differ, that they can hardly be supposed to refer to the same disease, the one representing it to be very mild, and the other as directly the reverse.

In 1735, it made its appearance in New England, gradually diffusing itself, to a greater or less extent, over this continent. Much confusion, however, exists in the history of the disease, owing to the want of a just discrimination in those by whom it is described, and especially at an early period. No distinction is made in most of these accounts between it and measles, or even small-pox, except as to gradation of violence,—and, so lately as the time of Moreton, and even of Watson, the latter of whom wrote about the middle of the last century, the identity of it and measles was maintained. Bateman declares " that Withering's publication on it in 1778, or rather the second edition of it in 1793, may be considered as the date of the correct diagnosis of the disease."

No inconsiderable share of attention has scarlatina occupied, and to which it is entitled, being frequent in its recurrences, extensive in its prevalence, and often exceedingly fatal in its ravages.

Nosologists divide it into three forms, which, however, are to be regarded as the same disease, presented in different aspects. In this state of subdivision, it has received the denominations of scarlatina simplex, scarlatina anginosa, and scarlatina maligna, the two latter sometimes also called cynanche maligna, or cynanche putrida, and the last, in our own language, malignant or putrid sore throat. It is unnecessary to enter into any discussion to establish the identity, except as to degree of severity, of these three forms. Cullen, I think, has satisfactorily effected this, when designing to prove the contrary. Endeavouring to show, that scarlatina anginosa, and cynanche maligna, are specifically different, he has proved their sameness. The several states of the disease, may prevail simultaneously even in the same family. Thus, when one child has scarlatina simplex, a second may be affected by scarlatina anginosa, a third with scarlatina maligna,—and hence, the common origin of these varieties, may be inferred. By the late Professor Gregory of Edinburgh, who had seen it, this has been stated, and I believe, that similar occurrences are not very uncommon, having met with them myself. The several grades of the disease are related to each other, as distinct and confluent small-pox, typhus mitior and gravior, or as autumnal fever in its several modifications. Discerning no more reason, therefore, for thus dividing scarlatina, than most other diseases, which are equally marked by gradations of violence, or occasional deviations in some prominent symptom, or affection, I shall disregard the accustomed arrangement, and treat of it as phlogistic or congestive, conceiving this to be a much more simple and correct distribution of the subject, and particularly as concerns practical convenience.

An attack of the first, or inflammatory description of the disease, is mostly preceded by gastric disturbance and præcordial anxiety, lassitude and weariness, some uneasiness of the head, and depression of spirits, chilliness, pains in the loins and limbs, followed by fever, with more than ordinary heat of surface, and acceleration of the pulse. Contemporary, or nearly so, with these phenomena, soreness of the throat, rigidity of the jaws, and difficulty of swallowing, are sometimes, though not invariably complained of, and where such exist, redness, and swelling of nearly the whole of the lining membrane of the fauces, may be perceived. These affections progressively increase as the case becomes more developed—the fever being higher, the pulse more vigorous and rapid, the pain of the head, especially in the direction of the frontal sinuses, aggravated, the temperature of the skin so raised as to amount, in some instances, to one hundred and eight, or even to one hundred and ten degrees of Fahrenheit, according to Currie and Willan. Nausea, or vomiting, or both,—considerable tenderness of the epigastrium, and more præcordial uneasiness, with jactitation and delirium, or tendency to it, are apt to take place, especially on the exacerbation in the evening, which commonly happens.

Examining the throat at this time, where the anginose state is prominent, the affection of it will be seen to have correspondently augmented, the velum pendulum palati, uvula, tonsils, and, in short, the whole of the fauces, more intensely florid and tumefied, which state extending up the Eustachian tubes, hearing is impaired, with often severe earache. Considerable as the difficulty of deglutition may be, it is not so great as might be supposed, and seems to be owing more to an affection of the muscles subservient to the process, than the throat itself. The tongue, which nearly from the beginning is furred, now presents clean polished edges, with a thick, tenacious coating of the root and centre, and in some instances, florid elongated papillæ projecting through the encrustation, resembling a sort of vegetation,—while, on other occasions, I have seen it perfectly clean, and of a fiery red appearance.

Commonly, the eruption appears at the close of forty-eight hours, though in the anginose cases, it is more delayed, and may be to double this period. In some cases, however, its occurrence is exceedingly sudden, with scarcely any premonition. It comes out first on the face and neck, thence successively on the trunk and extremities, and becomes very conspicuous about the loins, bendings of the joints, and on the hands and fingers, which are stiff and swollen. In the simple variety of the disease, especially, the eruption often appears also on the surface of the interior of the mouth, the palate and fauces, of the same speckled or punctated character as that of the exterior, by which circumstance, and the absence of tumefaction, it may be distinguished from the real anginose affection. As to the diffusiveness of the exantheme, there is great difference, sometimes it being nearly universal, and at other times partial, or in patches. It consists of a multitude of small points, at first of a pale or dingy red, gradually assuming the scarlet hue, which spread, and apparently coalesce, so as to exhibit a blush of a more or less extent, of so deep a colour as to "resemble that of a boiled lobster,"—the comparison used in one of the best descriptions of it. Yet carefully examined, the efflorescence will still be perceived to be made up of specks, shaded off, from the centre to the edges.

The skin continues hot, and is itchy, sore, and somewhat swelled. Not very sensibly influenced is the fever by the appearance of the exantheme; sometimes, however, it abates, with great relief to the stomach and head, though as often it is otherwise, and may be exasperated. It usually remains in some degree till the desquamation is completed, which may be very tardily performed. The eruption, as well as the affection of the throat, is also very much controlled by the fever, which early abating, each is moderated, and more speedily subsides. But where it is maintained, the reverse happens, and the throat especially, becomes worse. The swelling is greater, and on its surface apthæ may appear, sometimes ragged and irregular, soon degenerating into ash-coloured sloughs, or an exudation of lymph takes place, in small portions, or so extensively, as to form an adventitious pellicular covering. In some cases, the inflammation, and its consequences, spread to the windpipe, productive of a variety of croup. But the result promising to be favourable, the sloughs, as well as the membrane, separate and are detached about the eighth or tenth day, leaving the surface beneath clean and healthy, and convalescence is confirmed.

What has been said is applicable to an average of the inflammatory state of the disease. But it is sometimes infinitely milder, and with little or no affection of the throat,—while, on other occasions, it presents a far graver and complicated aspect, all the symptoms, general and local, being highly exasperated.

Congestive scarlatina, though retentive of the common characteristics of the disease, these receive the complexional hue, which is always bestowed by the operation of the typhoid influence, or condition. Commencing with many of the initiatory phenomena noticed under the preceding head, the peculiarities of this mode of attack are soon manifested. For the most part, reaction is exceedingly feeble or imperfect, and may not at all take place. The collapsed state is marked by a weak pulse, cold skin, doughy countenance,—the head rather stupid than aching,—the stomach dreadfully distressed, the respiration laboured, with deep sighing,—the whole attended by extreme debility, and a disposition to syncope. It may, however, happen, that after a while, the system emerges from under such heavy oppression, or it being originally less, some vigour is displayed, by a full, soft, compressible pulse, an intensely hot, dry surface, amounting even to the *calor mordax*, with pain in the head, suffused eyes, turgid countenance, tenderness of the epigastrium, or of a lower portion of the abdomen, accompanied by vomiting or purging, or both. But this excitement, usually evanescent, is succeeded by a sudden and alarming prostration of vital power.

Be the early stage as it may, the subsequent career of these cases does not materially vary. As they proceed, the typhoid condition becomes more confirmed. The tongue, at first heavily furred, is now thickly coated of a dark brown colour, at the root and middle, with the vegetations formerly mentioned, while the edges are red, clean, and dry. The face is bloated, and may be livid, the eyes fatuous as in inebriation, the intellectual faculties depraved by low delirium, attended by nervous tremors and automatic motions of the hands. Excepting the brain be deeply implicated, when the pulse is slow, it is very rapid and weak, and the stomach evidently suffers sometimes, without vomiting, its powers being so paralyzed, as it were, as to prevent it, though its contents, consisting of a dark flocculose fluid, are ejected by a sort of spasmodic effort of the diaphragm analogous to singultus. More generally in this modification of the disease, are the pulmonary organs involved. Early betrayed by cough and hoarseness, with defluxions from the eyes and nostrils, these are speedily followed by very embarrassed breathing, with wheezing and rattling from the immense secretion of vitiated mucus. In the state of excitement which has been noticed, though the eruption may come out very quickly, even earlier than in the inflammatory form of the disease, it is more frequently postponed to a longer period, alternately appears and recedes, is in patches of a pale, rapidly changing to a mahogany, or livid, or purple colour, forming the scarlatina purpura of writers,—in some instances becomes widely diffused, and in others, it never appears. The same may be said of the anginose affection. During the prevalence of the disease in the winter of 1834, in this city, more than a dozen cases came under my observation, and in one family three persons, whom I attended with Professor Jackson, without either affection. Each of them sunk in a few hours, under the oppression of the early or collapse stage. These cases were known to be scarlatina, from the disease, unequivocally developed, co-existing in the same house. Commonly, however, the throat seriously suffers, and very often independently of the skin. The epidemic to which I have just alluded, abounded in such examples, as well as of the contrary, or the efflorescence alone appearing.

In the character of the local affection, there is some difference from that of the inflammatory disease. Equally pervading, less tumefaction, however, prevails, and it is of a darker complexion. Much more apt, too, is the surface to be covered by a membrane. But here, it is usually thick, soft, and pultacious,—and, should apthous ulceration exist, this very rapidly and widely spreads, and becoming gangrenous, discharges an acrid, ichorous fluid, which passing out of the mouth or nose, or both, excoriates the parts it touches, and the *fœtor oris* is excessive. Concomitantly the voice is hoarser, the respiration extremely oppressed, deglutition more difficult, with increased rigidity of the jaws, and there is a constant though ineffectual effort to disengage and bring up the irritating matters. This state of things not being arrested, heavy stupor supervenes, with sometimes petechiæ or vibices, and passive hæmorrhage, or colliquative diarrhœa, the pulse so diminutive as scarcely to be felt, and finally death takes place from absolute exhaustion, if not more abruptly by some cerebral, laryngial, or pulmonary affection.

The duration of the disease, is from a few hours to ten or fifteen days, according to the nature of the attack, being in this respect subject to many deviations. Going through its career with regularity, and mildly, it will be mostly found, that on the fourth day it has attained its height, on the fifth the efflorescence begins to decline, on the sixth it has nearly faded, and on the seventh entirely gone, leaving the skin dry and scaly. In some rare instances, however, which have been recorded, there has taken place just in anticipation of the disappearance of the rash, an eruption more or less vesicular or pustular, sometimes resembling varicella, and on other occasions, variola so closely, as to have been denominated *scarlatina varioloides.* No such instance have I ever met with, and whether it be the consequence of the original disease, or a distinct affection developed on the subsidence of the preceding one, I cannot say.

From scarlatina generally prevailing as an epidemic, its production has been assigned to the occult and mysterious agency of such diseases. But however its propagation may be promoted or its character affected, by an influence of this kind, there can be little doubt that its immediate cause is a specific contagion, conforming in this and other respects to the diseases to which it is most closely allied. Exactly like these, it destroys the susceptibility to a second attack. By Withering and Willan, this is positively asserted, they never having witnessed an instance to the contrary,—and Bateman states, that the fact is fully ascertained and accredited. Nevertheless, the infallibility of the protection has been denied, and examples of failure are cited by respectable writers, among which is Richter, who avers that a second, and even a third repetition of attacks, have been noticed. Conceding this, it still rests on the same footing as variola, and other diseases of acknowledged contagiousness. It is questionable at what stage scarlatina acquires this property, prior or subsequent to the eruption. But many believe, that it is most active during the desquamation, and at all events, it seems to retain it till this process is completed, the scales being impregnated with the virulent secretion of the skin. Yet we are told that the disease cannot be propagated by inoculation with matter procured from this or any other source. Contrary statements have been made, I am aware, though resting on no good authority. To *fomites* of various kinds, it adheres very tenaciously for a long period, with an entire preservation of its efficiency, in proof of which we have some striking facts. Thus, my friend Dr. Percival, of Dublin, imputes the introduction of scarlatina into that city, to a box of toys from London, which had been exposed to the contagion. That the disease was propagated in this way, however, is hardly to be credited,—and the story seems to me very like the famous report of Hilderbrandt, who assures us that as soon as he arrived in Podolia, scarlatina broke out and spread most widely, which he ascribes to the retention of contagion in a coat he had worn in the disease a year previously in Vienna. Nevertheless, the long continuance of infection in apartments where the disease has existed, though every purification be practised, is unquestionable. It is stated by Professor Elliotson, in confirmation of it, that all the children admitted into a particular ward in a hospital under his care, were seized with scarlatina for nearly two years in consequence of a patient with the disease having been in the ward at that remote period, and this in despite of white-washing and other cleansings.

Extraordinary as this may appear, it is by no means incredible, and is supported both by some further facts and by analogies.

Not to refer to other instances, in the spring of 1834, I attended a boy in the disease, whose parents being exceedingly anxious that their children should escape it, had them all immediately sent away. Every article of the furniture of the room was removed, the carpets taken up, the bed emptied of its feathers, the ticking washed, and his clothes destroyed. Besides this, the freest ventilation was practised, and the fumes of the chlorate of lime filled the whole house for several days. Notwithstanding all these precautions, twelve weeks afterwards on the return of the children, three of them became speedily affected through the medium of some domestic fomites in all probability, as they were not elsewhere exposed to the contagion.

Nothing more occurred here, than has been reported again and again of variola, typhus, and even of puerperal fever. Five or six years ago, the latter broke out in the lying-in-ward of our hospital—and though every means of purification was adopted, no sooner was a woman delivered in the ward, than it reappeared, and in a very fatal shape. Each successive year for three terms, was the ward closed against admissions, the process of purification again repeated, and still on the re-introduction of parturient women, after such a protracted interval, the result was the same, and now the ward is abandoned.

As to the sphere in which the contagion of scarlatina operates, we have no precise information. But it may be presumed to be similar to that of variola and the other diseases of the same class, which you will recollect is very limited.

It is said that the incubative period of the contagion is five or six days. My own conviction is, that it is usually greater, though I speak diffidently on the point, having been unable to satisfy myself in regard to it. Equally subjected apparently to infection, I have seen individuals break out with the disease from the third to the eleventh day; and we have had some cases lately reported, where in a family of eight, the interval varied from seventeen to the twenty-sixth day, the average being seventeen days.

No season is exempt from scarlatina, though it is most apt to prevail in winter or spring. But I have seen it during the hottest weather, and indeed at all seasons. Children are chiefly liable to it, as they are to every similar affection, for no other reason probably, than that persons in more advanced life have previously had it. Yet, I have never known a very young infant to take it, however exposed. By Sir Gilbert Blane it is asserted, that he never saw an individual, except one, affected by it turned of forty. But I have met with it several times in people much older, and once with Dr. Dewees, in a very aged man near eighty. It has, too, been remarked as one of the peculiarities of the disease, that on some occasions, it is restricted to children, and on others to adults, and those a little more advanced. Thus Reil declares, that he had witnessed an epidemic scarlatina, which was almost exclusively confined to persons between fifteen and twenty-five.

The difficulty that may exist relative to the diagnosis of scarlatina, must arise chiefly from its occasional similarity to measles. Even here, embarrassment can seldom be experienced, should it be recollected, that in measles, the symptoms are more conspicuously opthalmic, catarrhal, or pneumonic; that the eruption comes out usually on the fourth day in blotches somewhat elevated, so that the surface does not exhibit a uniform blush, and that these blotches running into each other assume a crescent shape, the whole being of a faint reddish complexion: while, on the contrary, that scarlatina is preceded by gastric and cerebral derangement—that the eruption occurs in half the time, consisting of minuter specks, seldom at all raised, is infinitely more diffused, and of a scarlet colour. The case being of an anginose nature, all ambiguity ceases; for though the throat is sometimes sore in each disease, it is in a manner so different as not readily to be mistaken. Generally in measles, it may be perceived to be merely an extension of the exantheme of the surface to these parts. Much reliance may also be placed on the singular appearance of the tongue, and the extreme acceleration of the pulse in scarlet fever.

As bearing an analogy to scarlatina, it may, however, be also mentioned, that an efflorescence frequently with sore throat, is to be met with, especially in children, having the popular title of *scarlet rash.* It differs from it, however, in the first place, in being usually occasioned, obviously, by suppressed perspiration, or disordered stomach from an excess of food, or certain peculiarly offensive articles; and, secondly, by the speedy occurrence of the eruption, without much or any previous ailment, and is rather a blush or suffusion than speckled. But I have seen it, on several occasions, dotted like scarlatina, and owing to some unintelligible cause of a pretty wide prevalence, several of a family being attacked in rapid succession, and still further spreading through the community. This is probably a variety of roseola, which is not contagious, or gives any security against scarlet fever.

In simple scarlatina, little danger is ordinarily to be apprehended. Yet, in some instances, it most unexpectedly becomes alarming, when apparently it had been doing perfectly well, by a sudden sinking of vital energy, or by its being raised into an exasperated state with diverse complications. No disease, indeed, is more treacherous, or requires greater vigilance. Two children whom I attended in consultation on different occasions, died almost immediately after our leaving them,—though at the time of our visits, they appeared to be doing well,—the first in the early, and the second in the advanced stage of the disease.

The other varieties of scarlatina are always to be dreaded; and the malignant condition of it, especially when epidemically prevailing, is occasionally as fatal as the plague itself. Many instances of such mortality have been reported; and among others, is that of its memorable occurrence at Naples already referred to, and those subsequently described by Moreton, Huxham, De Haen, Sims, Fothergill, &c. We are, indeed, told that when it prevailed in Paris, in 1743, so indomitable did it prove, that not a single individual recovered, and numbers perished within the short space of a few hours. But, on the contrary, it sometimes appears epidemically in the character of extreme mildness, as recorded by Moreton, Sydenham, and other authorities.

Feeble reaction with heavy congestions, or intense fever, is unfavourable, though the former more so. Any material deviation in the efflorescence from its common character and order,—the eruption being too early or late,—or alternately coming out and receding,—or its appearance in blotches only,—or an entire absence of, or permanent repercussion of it,—or its having a pale or livid, or mahogany colour,—or its rapidly changing from the one to the other of these hues,—or if attended by petechiæ or vibices, are all of bad import.

It is very unfavourable to have the throat affected, without any eruption, it denoting a concentration of the disease on the internal organs, and especially when the lesions are extensive, and the aspect dark, with apthous ulcerations. Even more so, however, is the croupy affection of the windpipe, from which recoveries very rarely take place. Nor scarcely less to be dreaded is a deep implication of the nervous system, or a quick, irritated, and feeble circulation. Extreme cerebral, or laryngial, or pulmonary, or gastric affection,—a gangrenous state of the fauces,—diarrhœa of acrid matter, or copious discharges of pallid urine, with jactation and debility, and an irritated or very weak pulse, indicate indeed, uniformly, the most imminent peril, and mostly prove the immediate precursors of death. By Reil it is remarked, that a white streak passing down on each side of the nose, and encircling it below, is a mortal sign, which, however, I have not seen.

An attack indicates a happy issue, whatever may be the form of the disease, where the eruption comes out in due season, is widely spread, of a bright red, regularly passing through the several stages to desquamation, and the throat being affected, the tumefaction is considerable, and florid, with painful deglutition, and white instead of gray or dark sloughs, the whole attended by moderate inflammatory fever. Every thing, in short, depends on the degree of reaction, the want of which almost invariably proves fatal, and when present, a different result may as confidently be anticipated from skilful management.

